# Proteomic Study of Retinal Proteins Associated with Transcorneal Electric Stimulation in Rats

**DOI:** 10.1155/2015/492050

**Published:** 2015-03-04

**Authors:** Takashi Kanamoto, Nazariy Souchelnytskyi, Takuji Kurimoto, Yasuhiro Ikeda, Hiroaki Sakaue, Yasunari Munemasa, Yoshiaki Kiuchi

**Affiliations:** ^1^Department of Ophtahlomology, Hiroshima Memorial Hospital, 1-4-3 Honkawacho, Minami-ku, Hiroshima 730-0802, Japan; ^2^Personalized Cancer Medicine Unit, Department of Oncology-Pathology, Karolinska Institute, 171 77 Stockholm, Sweden; ^3^Department of Ophthalmology, Osaka Medical College, 2-7 Daigaku-cho, Takatsuki-shi, Osaka 569-8686, Japan; ^4^Department of Ophthalmology, Kyushu University, 3-1-1 Maedashi, Higashi-ku, Fukuoka 812-8592, Japan; ^5^Department of Pharmaceutical Sciences, International University of Health and Welfare, 2600-1 Kitakanemaru, Tahara-shi, Tochigi 324-8501, Japan; ^6^Department of Ophthalmology, St. Marianna Medical College, 2-16-1 Sugao, Miyamae-ku, Kawasaki-shi, Kanagawa 216-8511, Japan; ^7^Department of Ophthalmology and Visual Sciences, Hiroshima University, 1-2-3 Kasumi, Minami-ku, Hiroshiam 734-8551, Japan

## Abstract

*Background*. To investigate how transcorneal electric stimulation (TES) affects the retina, by identifying those proteins up- and downregulated by transcorneal electric stimulation (TES) in the retina of rats. *Methods*. Adult Wistar rats received TES on the left eyes at different electrical currents while the right eyes received no treatment and served as controls. After TES, the eye was enucleated and the retina was isolated. The retinas were analyzed by proteomics. *Results*. Proteomics showed that twenty-five proteins were upregulated by TES. The identified proteins included cellular signaling proteins, proteins associated with neuronal transmission, metabolic proteins, immunological factors, and structural proteins. *Conclusions*. TES induced changes in expression of various functional proteins in the retina.

## 1. Background

Electrical stimulation has been shown to enhance the regeneration of axons after surgical transaction of the femoral nerve in adult rats [1] and accelerate the speed of femoral motor axonal regeneration [2]. Thus, electrical stimulation can have regenerative effects on damaged peripheral nerves.

Transcorneal electrical stimulation (TES) has been tried on adult rats and human patients. In an animal model of retinitis pigmentosa, TES prolonged the survival of photoreceptors morphologically and delayed the decrease of retinal function by electrophysiological analysis [3]. TES also promoted the survival of retinal ganglion cells (RGCs) of rats after optic nerve injury [4]. Furthermore, the results of TES in cat eyes suggested that TES activated retinal neurons through vascular changes [5].

In its clinical aspects, TES has been found to improve the visual acuity and peripheral visual field in patients with traumatic optic neuropathy [6], and its single-time application to eyes with nonarteritic ischemic optic neuropathy (NAION) improved the subject's visual acuity and peripheral visual field [6]. It was reported that TES improved the visual acuity and visual fields in three patients with longstanding retinal artery occlusion [7] and that TES improved the visual field in eyes with branch retinal artery occlusion [8]. In addition, TES also improved the inner retinal function in patients with retinal degeneration, including retinal pigmentosa and cone-rod dystrophy [9], and also improved visual acuity in patients with vitelliform macular dystrophy [10]. Thus, TES has been proven to be beneficial for retinal neuronal diseases, including retinal vascular diseases and retinal degeneration, in clinical trials.

The mechanism by which TES alters the retinal neurons to lead to good outcomes has not been determined, however. This information is essential for justifying the use of TES to treat not only ischemic retinal diseases but also other types of retinal diseases. Retinal function is mediated by neural proteins, and neural proteins themselves are regulated by cellular protein signaling networks. So, we hypothesized that a proteomic analysis of the expression patterns of proteins induced by TES will provide evidence regarding the mechanism of retinal regeneration. Identification by proteomics of those proteins affected by TES would also have value for its own sake. We found that the expressions of 25 proteins were up- or downregulated after TES.

## 2. Methods

### 2.1. Animals

All experiments were performed in accordance with the principles of the Association for Research in Vision and Ophthalmology for the use of animals in ophthalmic research. The procedures used in these experiments were approved by the Animal Use Committee of Hiroshima University.

Adult Wistar rats were obtained from CLEA (Tokyo, Japan) and were housed in clear plastic cages containing pine bedding. The animal quarters were kept at 21°C on a 12 hr : 12 hr light : dark cycle.

### 2.2. Transcorneal Electrical Stimulation (TES)

The rats were anesthetized intraperitoneally with chloral hydrate. Only the left eye was electrically stimulated while the right eyes received no stimulation and served as the controls. For the stimulation, the cornea was anesthetized with a drop of 0.4% oxybuprocaine HCl, and a contact lens electrode with inner and outer circular concentric electrodes was placed on the cornea with a drop of 2.5% methylcellulose to maintain good electrical contact and prevent corneal dehydration. Biphasic rectangular (1 ms phase duration) current pulses were delivered at a frequency of 20 Hz from an electrical stimulation system (stimulator: SEN-8203, Nihon Kohden, Tokyo, Japan; isolator: A365, World Precision Instruments, Sarasota, FL) through the contact lens electrodes. The frequency was 20 Hz and the duration of the stimulation was 30 minutes, which had already been shown to be an appropriate time for stimulation [11]. The current intensity was varied at 50 *μ*A, 100 *μ*A, and 200 *μ*A. Only one session of TES was given, and the eyes were enucleated 30 minutes or 24 hours after the TES. Three rats were prepared in each group and, totally, eighteen rats were studied.

### 2.3. Two-Dimensional Electrophoresis and Gel Analysis

Rats were deeply anesthetized with chloral hydrate and the eyes were enucleated. The retinas from stimulated and nonstimulated eyes were carefully isolated in PBS and solubilized in sample buffer (8 M urea, 4% CHAPS, 0.5% DTT, IPG buffer, and pH 3–10). The protein concentration of lysatewas measured with the Bradford assay.

Two-dimensional electrophoresis and protein identification were performed as described in detail [12]. Isoelectrofocusing was performed on strips (pH 3–10 nonlinear gradient, 18 cm, GE Healthcare, Buckinghamshire, UK). The first-dimension isoelectrophoresis was performed in IPGphor (GE Healthcare) according to the manufacturer's instructions. After the isoelectric focusing, the strips were placed in equilibration buffer-1 (50 mM Tris-HCl, pH 8.8, 6.0 M urea, 2.0% SDS, 30% glycerol, and 1% DTT) and then in equilibration buffer-2 (50 mM Tris-HCl, pH 8.8, 6.0 M urea, 2.0% SDS, 30% glycerol, and 4% iodoacetamide). The equilibrated strips were loaded onto SDS-containing 10% polyacrylamide gel, and SDS-PAGE was performed. After the electrophoresis, the gels were fixed in 7.5% acetic acid and 20% methanol and sensitized in 25% ethanol, 0.2% sodium thiosulfate, and 3.4% sodium acetate. The gels were stained with 0.25% silver nitrate and developed with 2.5% sodium carbonate and 0.04% formaldehyde. Silver-stained gels were scanned by an image scanner (ES-2200, EPSON, Tokyo, Japan), and the volume of spots was determined with PD-Quest software (Bio-Rad Laboratories, Hercules, CA, USA) following the manufacturer's instructions. The values of the volume of each matched spot on the master gels were compared. Spots with differences in expression were then identified by mass spectrometry.

### 2.4. Protein Identification

The stained protein-containing spots were destained with 30 mM potassium ferricyanide and 100 mM sodium thiosulfate. Then, the gel pieces were dipped in 0.1 M sodium hydrocarbonate and washed with acetonitrile. After drying, in-gel digestion was performed with trypsin. Then, 0.1% trifluoroacetic acid (TFA) and 10% acetonitrile in water were used to extract the peptides, and the extract was desalted on a nanocolumn. After washing the column with 0.1% TFA in water, the matrix was eluted with acetonitrile containing alpha-cyano-4-hydroxycinnamic acid directly onto the MALDI target. Spectra were generated on a MALDI-TOF mass spectrometer (Bruker Daltonics, Billerica, MA, USA). The spectra were internally calibrated using known internal tryptic autodigestion peptides and searches were made in the NCBI database using Profound.

## 3. Results

### 3.1. Two-Dimensional Proteomic Maps of Rat Retinas after TES

To identify the proteins expressed after TES, we compared the proteomes in TES-stimulated and nonstimulated rat retinas. The total lysates of the retina at each current intensity (50 *μ*A, 100 *μ*A, and 200 *μ*A) and post-TES times (30 minutes and 24 hours after TES) were resolved by two-dimensional gel electrophoresis.

**Figure 1:**
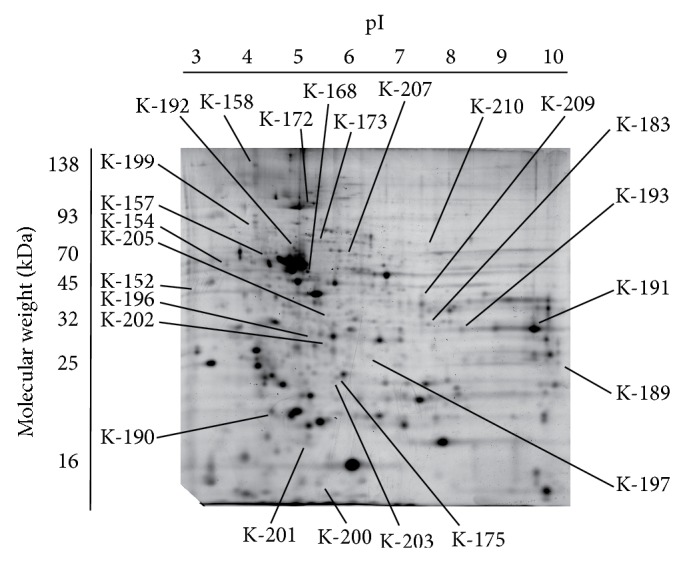
Photographs of a two-dimensional electrophoresis gel with annotation of the spots of identified proteins. The image shows a silver-stained gel of rat retina after TES. The proteins spots that were increased or decreased with or without TES that were identified by PMF are shown. Spots K-152 to K-210 represent the annotated spots. The pH gradient of the first dimension electrophoresis is shown at the top of the gels, and the migration of the molecular mass markers for SDS-PAGE in the second dimension is shown at the side of the gel. Representative gel images are shown.

We detected approximately seven hundred protein spots on the two-dimensional gels after silver staining. The volume of each protein spot was quantified by PD-Quest software, and the largest volume observed was 41,964 units. We analyzed three gels for each current intensity and post-TES time to ensure the repeatability of the protein volume measure. Thirty-seven protein spots, which were not detected without TES, remained as inducible expression by TES, and we could not see the protein spots whose volume decreased after TES. In addition, the pattern of expression changed between 30 min and 24 h after TES. We also performed a manual control of the staining quality and matching of spots directly on the stained gels. In the end, twenty-five spots that had significant upregulation of volume were selected (Figure 1).

### 3.2. Clustering of Identified Protein Spots

MALDI-TOF mass spectrometry was performed to identify the twenty-five proteins. All of them were successfully identified with high-quality spectra and probability scores of identification of *P* < 0.05.

Five of the 25 proteins, DLP-1, vimentin, angiopoietin-3, Elk, and ankyrin, were expressed only during the early phase, 30 minutes after the TES, while 10 proteins, immunoglobulin heavy chain 1a, SERPIN, calcineurin-2 regulator, Ras-related GTP binding B, Ca^2+^/Mg^2+^ ATPase, tenascin-X, EGF receptor, adenylate cyclase 10, TGF-*β* regulator 4, and dihydroxyacetone phosphate acryltransferase, were induced only in the latent phase. Others were expressed in both the acute and latent phases. These findings indicated that the effect of TES was still present 24 hours after stimulation and not just immediately after the stimulation (Table 1).

## 4. Discussions

Twenty-five proteins were differentially expressed in the retina of rats after TES, and their expression pattern was either early or latent after TES. TES after optic nerve transection in adult rats increased the number of surviving axotomized RGCs* in vivo* by increasing the level of insulin growth factor- (IGF-) 1 production by Muller cells [13]. An increase in brain-derived nerve growth factor (BDNF) expression at the mRNA and intracellular protein levels has been found in cultured Muller cells after TES [14]. TES also increased the expression of fibroblast growth factor- (FGF-) 2 [15]. However, neither IGF-1, BDNF, nor FGF-2 was upregulated in our proteomics results. This may be because our experimental model was different from previous ones in employing wild eyes without a crushed optic nerve.

The differentially expressed proteins included those involved in different kinds of cellular functions as follows. SIAH-2, adenylate cyclase 10, Ca^2+^/Mg^2+^ ATPase, calcium-binding domain 1, DLP 1, and calcineurin-2 regulator represent physiological factors identified as being differentially expressed. Nine cellular signaling molecules, GDI-2, angiopoietin-3, EGF receptor, Elk, Ras-GTP binding B, rho-GTPase activating protein, TGF-*β* regulator 4, prohibitin, and SERPIN, were identified. Three metabolic proteins were also identified, for example, HPPD, guanine nucleotide binding protein, and dihydroxyacetone phosphate acryltransferase. Two immunological proteins, HPPD and lymphocyte protein 1, and five structural proteins—vimentin, tenascin-X, VIF, keratin-8, and ankyrin—were upregulated by TES. These indicated that TES had effects on various kinds of cellular functions in the retina.

Among the identified proteins in this study, the pattern of protein expression was different at 30 minutes after TES and 24 hours after TES. While fifteen proteins were expressed at 30 minutes, 20 proteins were expressed at 24 hours, and, of these 20, 10 had not been expressed at the acute phase. Sergeeva et al. mentioned that transcorneal alternating current stimulation induces EEG “after-effects” only in rats with an intact visual system but not after severe optic nerve damage [16]. The difference in our results indicated that TES induces acute and chronic changes in protein expressions. It is suspected that the acute change is a direct and transient effect induced by electric shock to neural cells while the chronic change is an indirect and secondary effect in mRNA expression or the protein signaling pathway.

We found that several neuronal synaptic agents were included among the identified proteins with an after-effect (i.e., appearing only at 24 h, not at 30 min). SIAH-2 (K-183), a ubiquitin ligase enzyme, binds the major synaptic vesicle protein synaptophysin and facilitates its degradation by the ubiquitin-proteasome pathway [17]. GDI-2 (K-173) is associated with the plasticity of neurotransmission [18]. GDI-2 is a signaling protein that regulates the GDP-GTP exchange reaction with members of the Rab proteins involved in vesicle transport. This process is critical for the release of synaptic vesicles [19]. These findings suggested that TES may have an effect on synaptic transmission in the retina to lead to upregulation in retinal function.

Proteins with chronic change or an “after-effect” are more important than proteins with acute change only, because ophthalmologists seek the stable upregulation of retinal function from the clinical use of TES. The identified proteins with chronic change included several proteins related to Ca^2+^ regulation. The regulator of calcineurin-2 (K-196) determines intracellular Ca^2+^ levels through the GABA-A receptor [20]. Calcium-binding and coiled coil domain 1 (K-154) is related to Ca^2+^ regulation in mitochondria [21], and Ca^2+^/Mg^2+^ ATPase directly regulates intracellular Ca^2+^. These findings indicate that TES may affect intracellular Ca^2+^ regulation in retinal neural cells and increase retinal function.

Furthermore, some neuronal regenerative factors in after-effect proteins were induced by TES. EGFR (K-158) regulates the axonal regeneration [22], and prohibitin (K-175) is also associated with axonal regeneration [23]. Transforming growth factor (TGF) regulator 4 is in the TGF-beta family and mediates the generation of the nervous system [24], and adenylate cyclase isoform (K-172) also regulates neural network wiring [25]. Tenascin-X (K-201) is associated with neuronal development [26] and is reexpressed in adults during normal processes such as nerve regeneration. It has also been stated that tenascin-X is present in the optic nerve and in peripheral nerves at the time of axonal growth [27]. Ras-related GTP binding B (K-197) and rho-GTPase activating protein 24 (K-205) are also associated with dendritic regeneration [28]. Thus, EGFR, prohibitin, TGF regulator 4, adenylate cyclase isoform, tenascin-X, Ras-related GTP binding B, and rho-GTPase activating protein 24 are suspected to play roles in retinal regeneration after TES.

As known, a flicker light stimulation is often performed as one of the clinical examinations to stimulate the retina. The stimulation system in this study, electric stimulation with 20 Hz, was different from a flicker 30 Hz light stimulation. Though 20 Hz was the best condition for neuroprotection in the retina [5, 11], we should check differences in protein expression changes for various conditions of TES, that is, frequencies, in a future study.

In conclusion, TES had effects on the expression of retinal proteins. These results will contribute to our knowledge on the mechanism of how TES affects the retina.

## Figures and Tables

**Table 1:** Differentially expressed proteins by proteomics from the retinas with/without TES. K-152 to K-210 represent ID number of spots and sequence coverage, and the theoretical values of pI and Mr were obtained from the Profound search. The calculations of the experimental pI and Mr were based on the migration of the protein on a 2D gel. The expression changes present in the rat retinal protein volume ratio after 50 mA, 100 mA, and 200 mA currents of TES for 30 minutes and 24 hours after TES. “o” in expression means that the expression was undetectable in the PD-Quest-based analyses.

Number	Protein	Protein ID	Gene ID	Probability	*Z*	Sequence coverage (%)	Matched peptide	Mes. peptide	pI	MW (kDa)	Suspected pI	Suspected MW (kDa)	Ratio of protein volume after TES					
													30 min			24 hrs		
													50	100	200	50	100	200
K-152	Guanine nucleotide binding protein	NP_112298.1	gi 13591957	1.0*E* + 00	2.2	27	8	56	5.5	42.41	2–4	32–44	0	1.26 ± 0.86	0	0	0.91 ± 0.32	0.71 ± 0.59
K-154	Calcium-binding and coiled coil domain 1	NP_631929.1	gi 21070934	1.0*E* + 00	2.43	8	5	64	4.8	77.77	3–4.5	44–92	0.34 ± 0.59	0	0	0.34 ± 0.59	2.01 ± 2.47	0.6 ± 0.37
K-157	Keratin 80	NP_001008815.1	gi 57012388	1.0*E* + 00	0.9	14	6	50	5.9	51.05	3–5	44–92	0.77 ± 0.23	0.67 ± 0.29	2.58 ± 2.06	1.17 ± 0.15	1.04 ± 0.27	1.15 ± 0.26
K-158	Epidermal growth factor receptor	NP_113695.1	gi 25742617	1.0*E* + 00	1.21	7	7	20	6.7	138.3	3–5.5	92–200	0	0	0	0	0.83 ± 0.14	0
K-168	Dynamin-like protein DLP1 isoform DLP1-37	AAD31278.1	gi 4868358	9.7*E* − 01	0.59	19	10	49	7	80.79	4–6	32–92	0	0	0.84 ± 0.21	0	0	0
K-172	Adenylate cyclase 10	NP_067716.1	gi 11067413	7.9*E* − 01	0.4	10	12	54	6.6	188.29	4–6.5	67–140	0	0	0	0	1.31 ± 0.56	0
K-173	Rab GDI beta/GDI-2	CAA52412.1	gi 396433	1.0*E* + 00	0.87	18	5	22	5.7	51.18	4–6.5	44–92	0.99 ± 0.17	0.41 ± 0.54	0.66 ± 1.14	0	0	1.36 ± 0.83
K-175	Prohibitin	NP_114039.1	gi 13937353	1.0*E* + 00	2.12	32	8	61	5.6	29.86	4.5–7	24–44	0	0.65 ± 0.32	1.03 ± 0.53	0.78 ± 0.7	0	0.96 ± 0.95
K-183	SIAH-2/seven in absentia 2	AF389477_1	gi 19550385	9.1*E* − 01	0.57	13	3	21	6.3	29.47	6–8.5	32–67	0	0.58 ± 0.51	0	0.33 ± 0.34	0.33 ± 0.57	0
K-189	Immunoglobulin heavy chain 1a	P20761.1	gi 121055	9.9*E* − 01	1.14	19	5	28	8	37.11	8–11	24–44	0	0	0	0	0	1 ± 0.43
K-190	Serine protein inhibitor/SERPIN	AAL99574.1	gi 19850068	1.0*E* + 00	1.23	36	10	88	9.4	47.57	3.5–5.5	16–32	0	0	0	0	2.28 ± 1.97	1.17 ± 0.26
K-191	Transforming growth factor bets regulator 4	AAH87073.1	gi 56541114	1.0*E* + 00	1.04	16	9	70	8.9	71.62	8.5–10.5	32–67	0	0	0	0.41 ± 0.53	0.04 ± 0.04	0
K-192	Vimentin	NP_112402.1	gi 14389299	1.0*E* + 00	1.9	16	11	65	5.1	53.77	4–6	44–92	0	0	1 ± 0.52	0	0	0
K-193	Angiopoietin-like 3	AAH88192.1	gi 56971793	1.0*E* + 00	0.84	28	6	46	6.1	37.14	6.5–8.5	32–67	0	0	1 ± 0.73	0	0	0
K-196	Regulator of calcineurin-2	NP_783168.1	gi 28212228	9.5*E* − 01	0.65	9	2	13	5.4	27.64	4–6	24–44	0	0	0	0	0	1 ± 1.12
K-197	Ras-related GTP binding B	AAH78760.1	gi 50927585	1.0*E* + 00	2.43	15	5	49	6.1	43.61	5.5–8	24–44	0	0	0	0	0	1 ± 0.89
K-199	Ca^2+^/Mg^2+^ ATPase	AAA57270.1	gi 602486	1.0*E* + 00	1.36	11	4	43	5.5	57.01	3.5–5.5	44–92	0	0	0	0	0.79 ± 0.18	1.63 ± 1.28
K-200	Vimentin-type intermediate filament	NP_001001720.1	gi 48843733	1.0*E* + 00	1.66	30	3	16	6.4	18.88	4–6	0–24	0	0	2.8 ± 1.64	0	0.35 ± 0.57	0.77 ± 0.63
K-201	Tenascin-X	AAA91987.1	gi 841426	9.8*E* − 01	0.7	16	3	30	6.4	23.71	4.5–6.5	16–32	0	0	0	0	0.13 ± 0.63	1.48 ± 0.48
K-202	Hydroxyphenylpyruvate dioxygenase/F alloantigen	AAA40740.1	gi 202924	1.0*E* + 00	1.63	15	4	32	6.3	43.6	4.5–7	32–44	0	0.54 ± 0.42	0	0.69 ± 0.3	0	0.33 ± 0.58
K-203	Elk protein	CAA31777	gi 56095	9.8*E* − 01	0.73	6.6	2	25	6.6	43.05	4.5–7	24–44	0	1 ± 0.69	0	0	0	0
K-205	Rho-GTPase activating protein 24	AAH85797.1	gi 55249719	1.0*E* + 00	1.66	15	9	58	8.7	73.98	5–7	32–67	1.04 ± 0.29	0.58 ± 0.39	0.74 ± 0.4	0.34 ± 0.59	0	0
K-207	Ankyrin	AAB47551.1	gi 1841966	1.0*E* + 00	1.35	20	10	84	8.2	90.36	5–7	44–92	0	1 ± 0.13	0	0	0	0
K-209	Dihydroxyacetone phosphate acryltransferase	NP_445862.1	gi 16758146	1.0*E* + 00	1.09	31	14	106	8.2	77.74	6–9	32–67	0	0	0	1 ± 0.35	0	0
K-210	Lymphocyte cytosolic protein 1	AAH83855.1	gi 54035327	1.0*E* + 00	1.34	27	11	89	5.2	70.73	6.5–8.5	44–67	0	0	1.04 ± 0.88	0.71 ± 0.62	1.08 ± 0.85	0
